# Nanoenzymatic SERS bifunctional detection platform based on recognition competition strategy for ultrasensitive detection of diabetic retinopathy-related biomarkers

**DOI:** 10.3389/fbioe.2025.1623332

**Published:** 2025-07-18

**Authors:** Binbin Zeng, Xia Zong, Xinjue Dai, Lian Pan, Xiaowei Cao, Changhua Lu

**Affiliations:** ^1^ Jiangsu Key Laboratory of Integrated Traditional Chinese and Western Medicine for Prevention and Treatment of Senile Diseases, Medical College, Institute of Translational Medicine, Yangzhou University, Yangzhou, China; ^2^ Ophthalmology, The Affiliated Yixing Traditional Chinese Medicine Hospital of Yangzhou University, Wuxi, China; ^3^ Department of Endocrinology, The Affiliated Yixing Traditional Chinese Medicine Hospital of Yangzhou University, Wuxi, China; ^4^ Department of Pathology, The Affiliated Yixing Traditional Chinese Medicine Hospital of Yangzhou University, Wuxi, China

**Keywords:** nanoenzymes, surface-enhanced Raman scattering, recognition competition strategy, diabetic retinopathy, biomarker

## Abstract

Early detection and intervention in diabetic retinopathy (DR) are key to its prevention and treatment. In this study, we propose a surface-enhanced Raman scattering (SERS) bifunctional detection platform based on nanoenzymes catalyzing the tetramethylbenzidine (TMB) reaction, which innovatively introduces an aptamer recognition competition strategy and achieves an ultrasensitive detection of DR associated biomarker (VEGF). The platform employs Au@Pd nanorods (Au@Pd NRs) modified with single-stranded DNA1 (ssDNA1) as nanoenzymatic probes. Arrays of Au trioctahedra (Au TOHs) with surface-modified double-stranded structures, including aptamer strands and single-stranded DNA2 (ssDNA2), were used as capture substrates. When the target protein is present in the solution to be tested, the aptamer specifically recognizes the target protein and detaches from the surface of the capture substrate, exposing ssDNA2 and being recognized and bound by ssDNA1, allowing a large number of nanoenzymatic probes to be bound to the capture substrate, and the assay platform thus possesses excellent POD activity and SERS performance, being able to catalyze the generation of TMB with a strong SERS signal oxTMB. The platform demonstrated high detection performance, completing the assay within 14 min, with a low limit of detection (LOD) of 0.11 pg/mL. It maintained robust clinical performance even in complex serum samples, and the results were consistent with ELISA. This work offers a framework for constructing nanoenzyme-SERS bifunctional detection systems and introduces a new approach for biomarker detection.

## 1 Introduction

Diabetic retinopathy (DR) is the most common ocular complication of diabetes mellitus (DM) and has become the leading cause of vision loss among diabetic patients globally (Cheung et al., 2010; Kropp et al., 2023). Epidemiological data indicate that the global prevalence of DM is approximately 10%, with DR affecting up to 22% of those diagnosed (Saeedi et al., 2019; Teo et al., 2021). DR is characterized by inflammatory and neurodegenerative damage to the retinal microvasculature, triggered by sustained hyperglycemia. This results in both structural and functional compromise of the blood-retinal barrier (BRB) and ultimately leads to dysfunction of retinal vascular endothelial cells (Yang and Liu, 2022). A major challenge in managing DR lies in its insidious onset, as early stages often lack noticeable clinical symptoms, making systematic screening essential for timely diagnosis (Motz et al., 2020; Lin et al., 2021). Current clinical diagnostic approaches include fundoscopy, optical coherence tomography (OCT), fluorescein angiography (FFA), and optical coherence tomography angiography (OCTA)(de Barros Garcia et al., 2017; Schreur et al., 2022; Waheed et al., 2023). However, widespread population screening remains difficult due to the time-intensive procedures and financial burdens associated with these methods (Egunsola et al., 2021). Thus, the development of affordable and practical screening tools for DR is of significant clinical relevance.

In recent years, biomarkers have played an important role in the diagnosis and efficacy evaluation of DR. Vascular endothelial growth factor (VEGF) was found to be the most critical biomarker in the early diagnosis of DR (Ahuja et al., 2019). Under high-glucose conditions, the buildup of advanced glycation end-products (AGEs) and local hypoxia collectively drive VEGF upregulation. This elevation not only induces abnormal endothelial cell proliferation but also acts in concert with inflammatory mediators to significantly increase vascular permeability. VEGF plays a direct role in DR-related pathologies, including retinal exudation, hemorrhage, and aberrant neovascularization (Rodríguez et al., 2019; Liu et al., 2023). Moreover, in a study involving 116 participants (38 healthy controls and 78 DR patients), the serum VEGF assay yielded an area under the ROC curve (AUC) of 0.791, with diagnostic sensitivity and specificity reaching 92.3% and 94.7%, respectively (Ahuja et al., 2019), highlighting its clinical potential as a diagnostic marker. However, the typically low abundance of many biomarkers in body fluids such as serum, coupled with numerous interfering components, places stringent requirements on detection technologies.

Surface-enhanced Raman scattering (SERS) technique has become an important tool in modern biochemical analysis due to its excellent molecular fingerprinting ability and non-destructive detection properties (Nilghaz et al., 2022). SERS technology also exhibits excellent resistance to water interference, fast response times, and non-invasive benefits, making it uniquely valuable in the detection of complex body fluids (Zhang et al., 2019; Moisoiu et al., 2021). Its signal enhancement primarily relies on LSPR and charge transfer effects, with the degree of enhancement being closely tied to the presence of “hot spots” on metal surfaces formed by LSPR (Sun et al., 2020). Therefore, selecting substrate materials that generate abundant “hot spots” is critical for achieving high-sensitivity SERS detection. Au trioctahedra (Au TOHs) have attracted attention in recent years because of their uniquely sharp geometry. This nanomaterial is capable of generating highly localized electromagnetic field-enhancing “hot spots” in the tip and edge regions, which significantly enhance the SERS signal strength (Song et al., 2015; Zhao et al., 2024), and has become one of the ideal substrates for SERS detection. Studies have shown that precious metal nanoenzymes (e.g., Au, Ag, Pd, Pt, etc.), as a new type of nanomaterials with natural enzyme mimicry and strong stability, have great potential for use in bioassays due to their peroxidase (POD), oxidase (OXD), and catalase (CAT) activities (Chen et al., 2015; Cheng et al., 2020). However, while monometallic Pd shows strong catalytic activity, it produces weak SERS signals; Au demonstrates the opposite properties. By forming Au-Pd composite nanomaterials, the synergistic interaction between the metal components accelerates electron transfer and enhances the LSPR effect. This results in improved POD activity, better structural stability, and stronger optical and catalytic properties, which have shown broad potential in fields such as cancer treatment and anti-infection therapy (Zhang et al., 2016; Zhao et al., 2022; Luo et al., 2024; Tang et al., 2024). Therefore, by innovatively combining the application of SERS and the enzyme-like activity of nanoenzymatic materials and introducing an aptamer recognition competition strategy, a nanoenzymatic SERS bifunctional detection platform can be constructed, which can effectively improve the detection efficiency and sensitivity. Compared to conventional detection methods such as enzyme-linked immunosorbent assay (ELISA) (Dumestre-Pérard and Thielens, 2021), chemiluminescence immunoassay (CLIA) (Cao et al., 2022), and radioimmunoassay (RIA) (Bílek et al., 2017), this approach not only addresses the limitations of complex setups and stringent reaction conditions but also enables ultrasensitive and highly specific detection of trace biomarkers.

**FIGURE 1:**
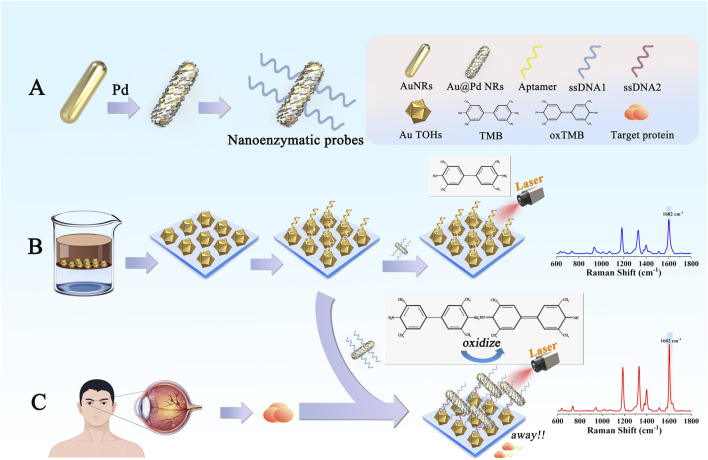
**(A)** Preparation process and functionalization of Au@Pd NRs. **(B)** Assembly and functionalization of Au TOHs. **(C)** The detection platform used in this study to detect biomarkers relevant to DR patients.

In this study, we developed a nanoenzymatic SERS bifunctional detection platform based on a competitive recognition strategy for highly sensitive detection of the DR-associated marker VEGF (Figure 1). This platform enables more direct screening of DR lesions through a distinct molecular recognition mechanism, showing a clear positive correlation between the detection signal and VEGF concentration. The experimental procedure was as follows: Au nanorods (AuNRs) were first synthesized using a seed growth method, followed by surface deposition of a continuous palladium shell to produce Au@Pd nanorods (Au@Pd NRs). Next, ssDNA1 modified with Au-S covalent bonds was modified on the surface of Au@Pd NRs as a nanoenzyme probe. To prepare the capture substrates, ordered arrays of Au TOHs were formed by self-assembly in the oil-water interface, and double-stranded structures, including aptamer strands (Aptamer) and single-stranded DNA2 modified with sulfhydryl groups (ssDNA2) were modified on their surfaces to obtain the capture substrates. The detection process involved three key stages. The first was the molecular recognition phase. Upon introducing a serum sample from a diabetic patient, VEGF specifically bound to the aptamer, while the nanoenzyme probe remained free in solution. During the competitive binding phase, VEGF bound to the aptamer with much higher affinity than the aptamer-ssDNA2 interaction, causing a displacement event. This step ensured high specificity and minimized interference from other serum proteins. In the final signal generation phase, the displacement allowed ssDNA1 to hybridize with ssDNA2 through base pairing, anchoring the Au@Pd NRs onto the surface of the capture substrate. This completed the integration of the nanoenzyme into the detection platform. At this stage, the platform utilized both the catalytic properties of the nanoenzymes and the SERS enhancement to catalyze the reaction of 3,3′,5,5′-tetramethylbenzidine (TMB) with H_2_O_2_, generating oxTMB with a strong SERS signal. By quantifying the SERS signal intensity of oxTMB, the VEGF concentration in serum could be accurately determined and compared to ELISA results to confirm the reliability of the method. This work introduced the catalytic activity of bimetallic nanoenzymes into the SERS assay for the first time using a competitive recognition strategy, and a nanoenzymes-SERS bifunctional detection platform was constructed, offering a novel approach for the early diagnosis of DR.

## 2 Experimental section

### 2.1 Materials and reagents

Chloroauric acid (HAuCl_4_), sodium borohydride (NaBH_4_), silver nitrate (AgNO_3_), hydrochloric acid (HCl), ascorbic acid (AA), sodium oleate, anhydrous ethanol, palladium chloride (PdCl_2_), bovine serum albumin (BSA), chloroplatinic acid (H_2_PtCl_6_), 5,5′-dithiobis (2-nitrobenzoic acid) (DTNB), and phosphate buffer solution (PBS) were purchased from Sinopharm Chemical Reagent Co. Cetyltrimethylammonium bromide (CTAB), cetyltrimethylammonium chloride (CTAC), tris(2-carboxyethyl)phosphine (TCEP), and TMB were purchased from Aladdin Biochemical Science and Technology Co. The nucleotide sequences listed in Table 1, including the VEGF aptamer and single-stranded DNAs (ssDNA1 and ssDNA2), were synthesized by Sangon Bioengineering (Shanghai) Co. The qRT-PCR kit was acquired from Getein Biotech (China). Deionized water (resistivity of 18.2 MΩ) was prepared using a Millipore purification system. Human serum samples were supplied by Yixing Hospital of Traditional Chinese Medicine.

**TABLE 1:** The experiment utilized nucleotide sequences listed in Table 1, read from 5′to 3′.

Name	Sequences
VEGF aptamer	TTT​TTT​TTT​TGT​GGG​GGT​GGA​CGG​GCC​GGG​TAG​A
ssDNA1	SH-AATCTACCCGGCCCGT
ssDNA2	SH-AAACGGGCCGGGTAGA

### 2.2 Material characterization

S-4800II Field Emission Scanning Electron Microscope (Hitachi, Japan), Tecnai12 Transmission Electron Microscope (Philips, Holland), Tecnai G2 F30S-TWIN Field Emission Transmission Electron Microscope (FEI, United States), Cary60 UV-Vis Spectrophotometer (Agilent, United States), and DXRxi Micro-Raman Spectrometer (ThermoFisher, United States).

### 2.3 Preparation of AuNRs

We optimized the experimental steps based on Tang et al. (2022). AuNRs with uniform morphology were synthesized using a seed-mediated growth method. Initially, 0.196 mL of HAuCl_4_ solution (25.4 mM) was mixed with 9.78 mL of deionized water, Then, 9.9 mL of CTAB solution (0.2 M) was added and mixed thoroughly. Next, 2 mL of freshly prepared NaBH_4_ solution (5.5 mM) was rapidly injected into the mixture under stirring at 1,100 rpm. Stirring was maintained for 120 s, during which the color changed from yellow to light brown. The solution was then left undisturbed for 1 h to complete seed formation. Subsequently, 500 mL of deionized water, preheated to 50°C, was used to dissolve CTAB (140 mM) and sodium oleate (26 mM). After cooling the solution to 30°C, 3.95 mM AgNO_3_ solution was added and allowed to react for 15 min. With continuous stirring at 800 rpm, 500 mL of HAuCl_4_ solution (1 mM) was introduced and stirred for 1.5 h until the solution became colorless. Then, 4.2 mL of 37% HCl solution (12.1 M) was added, followed by 2.5 mL of AA solution under stirring at 1,200 rpm. After 30 s, 0.8 mL of the previously prepared seed solution was added to the growth mixture, stirred at 1,300 rpm for 30 s, and then left undisturbed for 12 h. Finally, the product was collected by centrifugation at 9,000 rpm for 15 min, and the precipitate was redispersed in a 7 mM CTAB solution.

### 2.4 Preparation of nanoenzyme probes

Pd shells were formed on the surface of AuNRs through reduction in an aqueous solution containing CTAB and AA. A 10 mM H_2_PdCl_4_ solution was first prepared by dissolving 35.4 mg of PdCl_2_ powder in 2.4 mL of 0.2 M HCl using a 60°C water bath for 60 min, then diluted to a final volume of 20 mL with ultrapure water. To prepare the coating mixture, 4 mL of the 10 mM H_2_PdCl_4_ solution and 100 mM AA were added to 70 mL of 7 mM CTAB, followed by the addition of 80 mL of ultrapure water. The mixture was stirred vigorously for 5 min. Next, 3.4 mL of AuNRs solution was added to the prepared reaction mixture and stirred continuously at 25°C for 3 h. To remove excess CTAB, the resulting Au@Pd NRs were washed by centrifugation at 11,000 rpm for 15 min. The supernatant was discarded, and the precipitate was redispersed in 5 mL of ultrapure water. Subsequently, ssDNA1 (0.1 mM) activated by TCEP (1 M) was added to it and incubated for 2 h at 37°C. To block nonspecific binding sites on the particle surface, 15 μL of a 1 wt% BSA solution was added, and the mixture was incubated for an additional 1.5 h. Finally, the product was centrifuged at 12,000 rpm for 7 min to remove unbound reagents and nucleic acid strands, yielding the nanoenzyme probe.

### 2.5 Preparation of capture substrates

We prepared Au TOHs by improving the method described by Zhao et al. (2024). To begin, 19.5 mL of CTAB (0.1 M), 0.3 mL of HAuCl_4_ (10 mM), and 0.2 mL of H_2_PtCl_6_ (0.1 M) were mixed thoroughly. Then, 1.8 mL of freshly prepared ice-cold NaBH_4_ (10 mM) solution was added under low-speed stirring at 300 rpm. The mixture was stirred until it turned light brown with slight foaming. From this, 40 μL of the reaction mixture was combined with 20 mL of water to prepare a seed dilution solution for the next step. Next, 1 mL of HAuCl_4_ (10 mM), 10 μL of H_2_PtCl_6_ (10 mM), and 0.5 mL of AA (0.1 M) were added sequentially to 20 mL of CTAC (25 mM) and mixed well. Then, 1 mL of the prepared seed dilution was added, and the resulting solution was gently shaken and placed in an oven at 45°C for a static reaction lasting 7 min. A deep red solution indicated the successful formation of Au TOHs. To assemble the Au TOHs into ordered arrays, 2 mL of Au TOHs solution and 4 mL of n-hexane were added to a beaker, followed by 2 mL of ethanol. The mixture was left to stand for 120 s to allow the formation of an oil-water interface, where Au TOHs spontaneously self-assembled into a tightly packed, metallic film. This monolayer film was then transferred onto a silicon wafer previously treated with piranha solution and dried under constant temperature to form the Au TOHs array. For preparation of the capture substrates, ssDNA2 and aptamer were first hybridized in phosphate buffer (8 mM) containing MgCl_2_ (0.8 mM) and NaCl (20 mM). After incubation for 1.5 h, 800 μL of ssDNA2 (100 μM) was mixed with 80 μL of TCEP (1 M) and left to react at room temperature for 60 min to activate the sulfhydryl groups. The activated duplexes were then dropwise added onto the Au TOHs arrays and incubated at 37°C for 2 h to allow full attachment to the surface. Finally, the modified arrays were washed thoroughly with PBS and deionized water, completing the fabrication of the capture substrates.

### 2.6 Collection and processing of clinical samples

This study was approved by the Ethical Review Committee of Yixing Hospital of Traditional Chinese Medicine (Ethics Number: 2024-KY-054). Serum samples were collected from 20 patients diagnosed with DR and 20 healthy individuals admitted to the hospital. Participant selection was conducted in accordance with the criteria outlined in the Chinese Guidelines for the Prevention and Control of Type II Diabetes Mellitus (2020 edition), and all participants signed informed consent voluntarily. The exclusion criteria included individuals with concurrent eye diseases such as glaucoma or cataracts, those with type I diabetes, acute diabetic complications including ketoacidosis or hyperosmolar coma, severe organ dysfunction, autoimmune disorders, or recent retinal photocoagulation treatment. Collected samples were processed aseptically and sealed according to standard procedures before being stored at −80°C in an ultra-low temperature environment. All steps complied with laboratory regulations for cold-chain management and testing operations.

### 2.7 SERS measurements

Patient serum samples and nanoenzyme probes were added dropwise to the SERS detection platform. VEGF present in the serum initiated the competitive recognition process, allowing more nanoenzyme probes to bind to the capture substrate. After incubation, unbound probes were washed off with PBS buffer and deionized water. A reaction mixture containing TMB (1.0 mM) and H_2_O_2_ (0.6 mM), adjusted to pH 4.5 using sodium acetate buffer, was then added dropwise to the platform. The reaction was conducted at 30°C for 14 min in a constant-temperature environment. When the solution turned visibly blue, SERS spectra were recorded using a laser with a wavelength of 785 nm, an exposure time of 10 s, and a power of 5 mW.

### 2.8 Data analysis

The sensitivity of the platform was assessed by determining the Limit of Detection (LOD), defined as the lowest reliably distinguishable concentration of the analyte. The LOD was calculated using the following formula:

LOD=3SD/K

where K is the slope obtained from linear regression of the signal-concentration curve, and SD is the standard deviation of the SERS intensity at 1,602 cm^−1^ for the blank sample.

## 3 Results and discussion

### 3.1 Characterization of Au TOHs arrays

**FIGURE 2:**
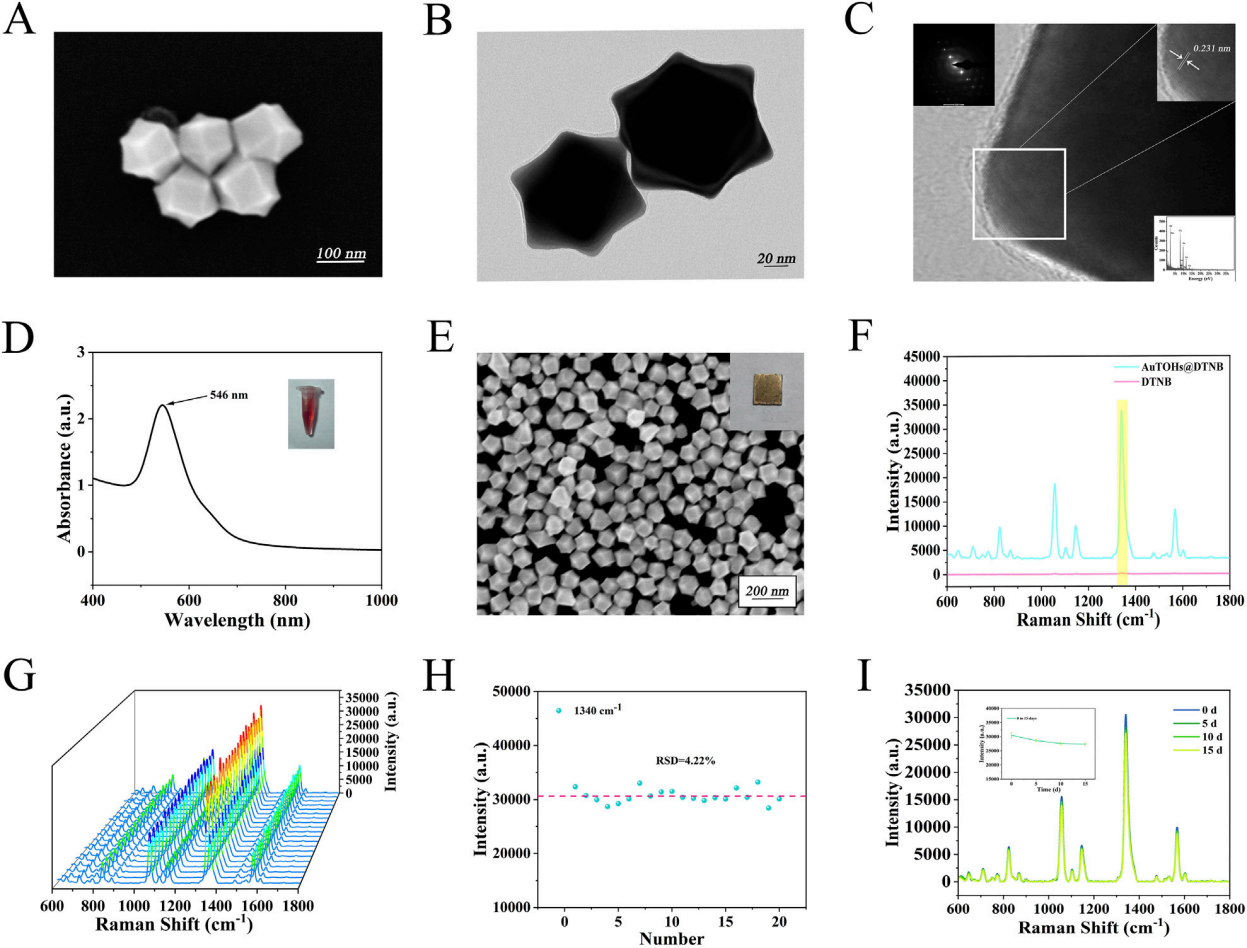
**(A)** SEM images of Au TOHs. **(B)** TEM image of Au TOHs. **(C)** HRTEM and SAED images of Au TOHs. **(D)** UV-Vis absorption spectra and solution appearance of Au TOHs. **(E)** SEM image of ordered Au TOHs array. **(F)** SERS spectra of DTNB (10 nM) labeled Au TOHs arrays and pure DTNB (10 mM). **(G)** SERS spectra at 20 randomly selected points on the DTNB labeled array. **(H)** Scatter plot of signal intensity at 1,340 cm^-1^ across different positions. **(I)** SERS spectra and intensity changes at 1,340 cm^-1^ after 0, 5, 10, and 15 days of storage.

Au TOHs with strong SERS activity were prepared as substrates for the detection platform. The addition of H_2_PtCl_6_ to the seed and growth solutions effectively enhances the purity and monodispersity of Au TOHs with negligible effects on the gold nanostructures. Pt selectively deposits on the high-energy crystalline surfaces, thereby inhibiting their growth without doping into the gold nanostructures (Zhao et al., 2024). Figures 2A,B present the SEM and TEM images of the synthesized Au TOHs, confirming their regular morphology, uniform dispersion, and sharp edge features. Figure 2C displays the HRTEM and SAED images, showing clear lattice fringes with a spacing of approximately 0.231 nm, indicating a single-crystalline structure. The UV-Vis-NIR absorption spectrum in Figure 2D reveals a prominent absorption peak near 546 nm. Figure 2E shows that Au TOHs formed tightly packed, ordered arrays via self-assembly at the oil-water interface. In addition, Au TOHs labeled using DTNB (10 nM) were compared with pure DTNB (10 mM) Figure 2F according to Eq: $EF=\left(\frac{I_{SERS}/C_{SERS}}{I_{Raman}/C_{Raman}}\right)$ , the value of EF was 1.88 × 10^9^, indicating that the Au TOHs array had a significant SERS enhancement effect. Figure 2G shows the SERS spectra collected from 20 randomly selected positions on the DTNB labeled Au TOHs array, indicating high signal uniformity. The scatter plot in Figure 2H represents the intensity values at 1,340 cm^−1^ across these positions, with a relative standard deviation (RSD) of 4.22%, further confirming the reproducibility of the array. In terms of stability, Figure 2I presents the SERS spectra of DTNB labeled Au TOHs arrays stored at room temperature over 0, 5, 10, and 15 days. A gradual decrease in signal intensity was observed, with an overall drop of about 10.11% compared to the initial state, suggesting that the arrays retain good stability over time.

### 3.2 Characterization of Au@Pd NRs

**FIGURE 3:**
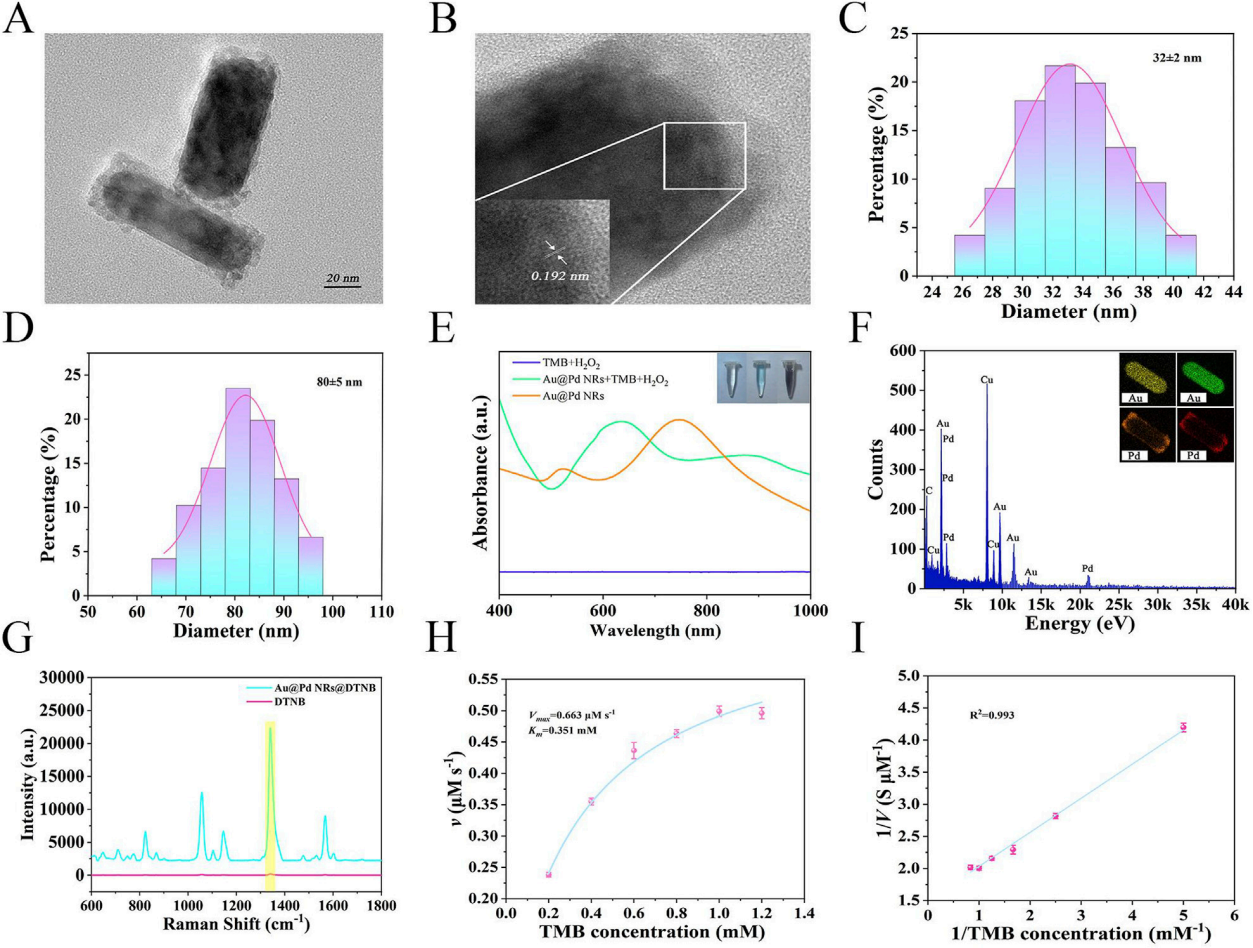
**(A)** TEM image of Au@Pd NRs. **(B)** HRTEM image of Au@Pd NRs. **(C)** Width distribution of Au@Pd NRs. **(D)** Length distribution of Au@Pd NRs. **(E)** UV-Vis spectra and color changes during sample catalysis. **(F)** EDX and Element Mapping for Au@Pd NRs. **(G)** SERS spectra of DTNB labeled Au@Pd NRs compared to pure DTNB. **(H)** Michaelis-Menten curve for Au@Pd NRs nanoenzyme. **(I)** Lineweaver-Burk plot for Au@Pd NRs nanoenzyme.

The transmission electron microscopy image in Figure 3A shows that the synthesized Au@Pd NRs exhibit uniform rod-like morphology with consistent size and shape. As seen in the HRTEM image in Figure 3B, the nanorods display a Pd-coated Au core-shell structure, featuring a rough surface and lattice fringe spacing of 0.192 nm. The average length and width of the Au@Pd NRs are approximately 80 nm and 32 nm, respectively, giving an aspect ratio of around 2.5:1, as illustrated in Figures 3C,D. The UV-Vis-NIR absorption spectra in Figure 3E show that the Au@Pd NRs solution has a distinct peak at 747 nm. When TMB was used as the substrate and in the presence of H_2_O_2_, Au@Pd NRs could catalyze the oxidation of TMB to produce a blue product and showed a strong absorption peak at 646 nm, whereas the solution was unchanged when TMB and H_2_O_2_ were present alone, which demonstrated its good catalytic activity. Elemental composition analysis through EDX and mapping (Figure 3F) confirmed that Au and Pd are the primary components of the nanorods. The detected Cu signal originates from the supporting TEM grid. As shown in Figure 3G, SERS spectra of DTNB labeled Au@Pd NRs were compared with those of pure DTNB (10 mM). The Au@Pd NRs displayed a strong SERS enhancement, with an enhancement factor (EF) calculated as 9.9 × 10^8^. To evaluate the catalytic activity of the nanoenzymes, we analyzed the Michaelis-Menten kinetics of Au@Pd NRs by fixing the H_2_O_2_ concentration, adjusting the TMB concentration, and setting up three replicate wells for each group (Figures 3H,I). The maximum reaction rate was determined to be 0.663 mM s^−1^, and the Michaelis constant (Km) was 0.351 mM for the Au@Pd NRs nanoenzyme.

### 3.3 Optimization of experimental parameters

**FIGURE 4:**
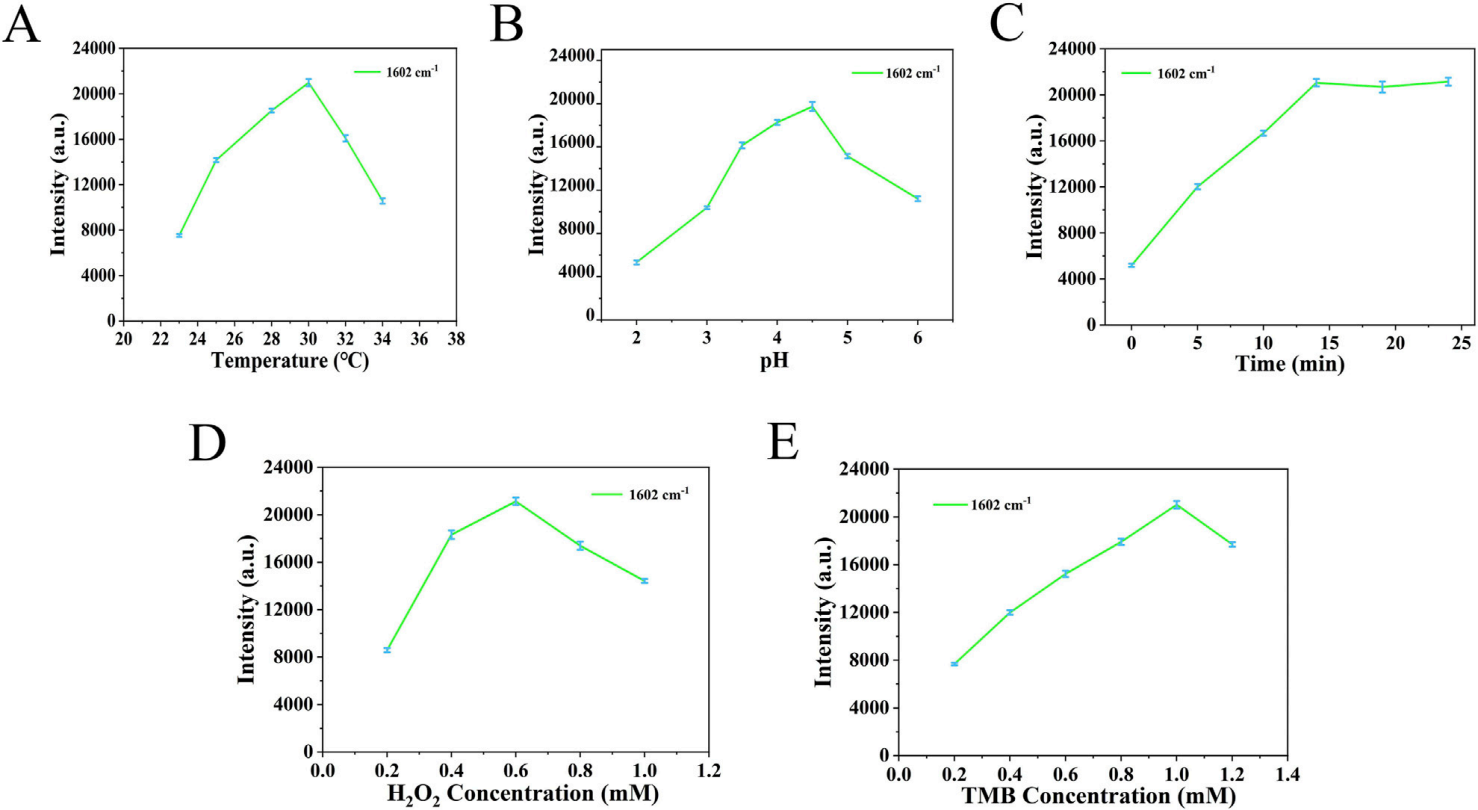
**(A)** Temperature parameters were optimized based on the signal strength at 1,602 cm^−1^. **(B)** PH parameters were optimized based on the signal strength at 1,602 cm^−1^. **(C)** The time parameters were optimized based on the signal strength at 1,602 cm^−1^. **(D)** H_2_O_2_ concentration was optimized based on the signal intensity at 1,602 cm^−1^. **(E)** TMB concentration was optimized based on the signal intensity at 1,602 cm^−1^.

During the target recognition process using the nanoenzymatic SERS bifunctional detection platform, both the pH and substrate concentrations significantly influence nanoenzyme activity. To ensure reliable testing, this study optimized key parameters using five detection platforms from the same batch. As shown in Figures 4A,B, the SERS signal intensity increased initially with rising temperature and pH, peaked, and then declined. The optimal conditions were determined to be a temperature of 30°C and a pH of 4.5. Figure 4C shows that the SERS signal intensity increased steadily over time, with the rate of increase slowing and reaching a plateau after 14 min. Therefore, 14 min was selected as the optimal incubation time. As indicated in Figures 4D,E, the highest SERS signal intensity was observed at a TMB concentration of 1.0 mM and an H_2_O_2_ concentration of 0.6 mM.

### 3.4 Performance evaluation

**FIGURE 5:**
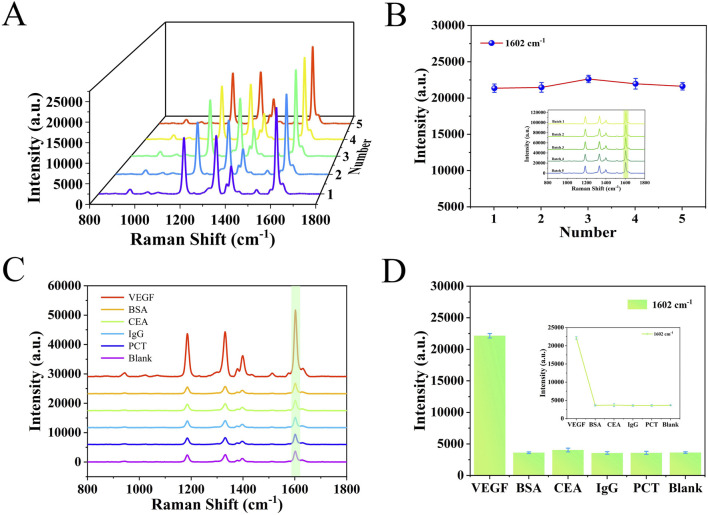
**(A)** SERS spectra from five different batches of detection platforms. **(B)** Line graph corresponding to the signal intensity at 1,602 cm^−1^. **(C)** SERS spectrograms obtained by detecting different analytes such as VEGF, BSA, IgG, CEA, PCT and blank. **(D)** Histogram of SERS signal intensity at 1,602 cm^−1^ characteristic peak.

The specificity and reproducibility of the detection platform were assessed under optimized conditions. As shown in Figures 5A,B, SERS spectra of five groups of VEGF were measured using independently prepared five-batch detection platforms. The RSD was 2.34%, indicating strong reproducibility and minimal variation across batches. To assess specificity, the platform was tested against structurally unrelated proteins, including three sets of the same concentrations of BSA, IgG, CEA and PCT. The results, shown in Figures 5C,D, revealed strong SERS signals only in the presence of VEGF. In contrast, signals for other analytes closely resembled the blank, confirming that these interferents did not affect detection. Furthermore, nonspecific binding was minimized by pretreating serum samples to remove non-target molecules and by thorough PBS rinsing of the platform. These measures supported the high specificity and reproducibility of the SERS detection platform developed in this study.

### 3.5 Quantitative testing

**FIGURE 6:**
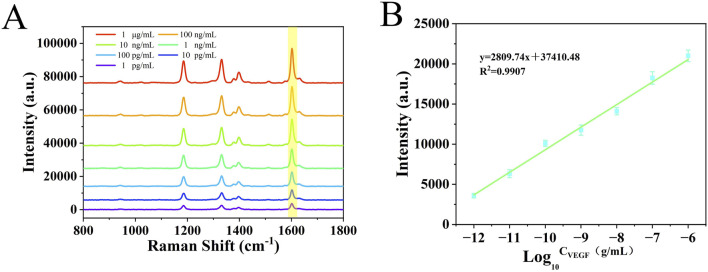
**(A)** SERS spectra of serum samples with different concentrations of VEGF (10 pg/ml^−1^ μg/mL). **(B)** Calibration curve of signal intensity *versus* logarithmic VEGF concentration at 1,602 cm^−1^.

Under optimized experimental conditions, the nanoenzymatic SERS bifunctional detection platform was applied to detect and quantify VEGF in serum across a range of concentrations. In order to accurately determine the lower limit of detection of this assay platform, in this study, we adjusted the concentration of healthy human serum samples to 1 mg/mL by pretreatment and addition of VEGF, followed by a gradient dilution method, adjusting the concentration of samples to 10 pg/ml-1 μg/ml. As shown in Figure 6A, with increasing VEGF levels, more Au@Pd NRs were recruited to the detection platform, leading to a progressive rise in the SERS signal intensity of catalytically generated oxTMB. This change in signal intensity corresponded to the concentration of the target protein. A linear regression curve was generated between the logarithm of the VEGF concentration obtained and the SERS intensity at 1,602 cm^−1^ by performing 3 sets of SERS assays for 7 different gradients of VEGF concentration (Figure 6B). The LOD was calculated to be as low as 0.11 pg/mL. When compared to previously reported detection strategies (Table 2), the method described here exhibits a substantially lower detection limit and strong specificity, enabling accurate measurement of VEGF in complex serum samples.

**TABLE 2:** Comparison of the methods in this study with other biomarker detection techniques.

Method	Target	LOD	Ref.
Fluorescence	PSPB	0.6 ng/mL	Hussain et al. (2024)
Electrochemistry	CEA	0.08 ng/mL	Khonyoung et al. (2024)
Fluorescence	Mb	4.99 μg/mL	Xu et al. (2024)
SERS	cTnI	9.09 pg/mL	Xiang et al. (2025)
Nano-enzymatic SERS	VEGF	0.11 pg/mL	This work

### 3.6 Clinical sample characterization

**FIGURE 7:**
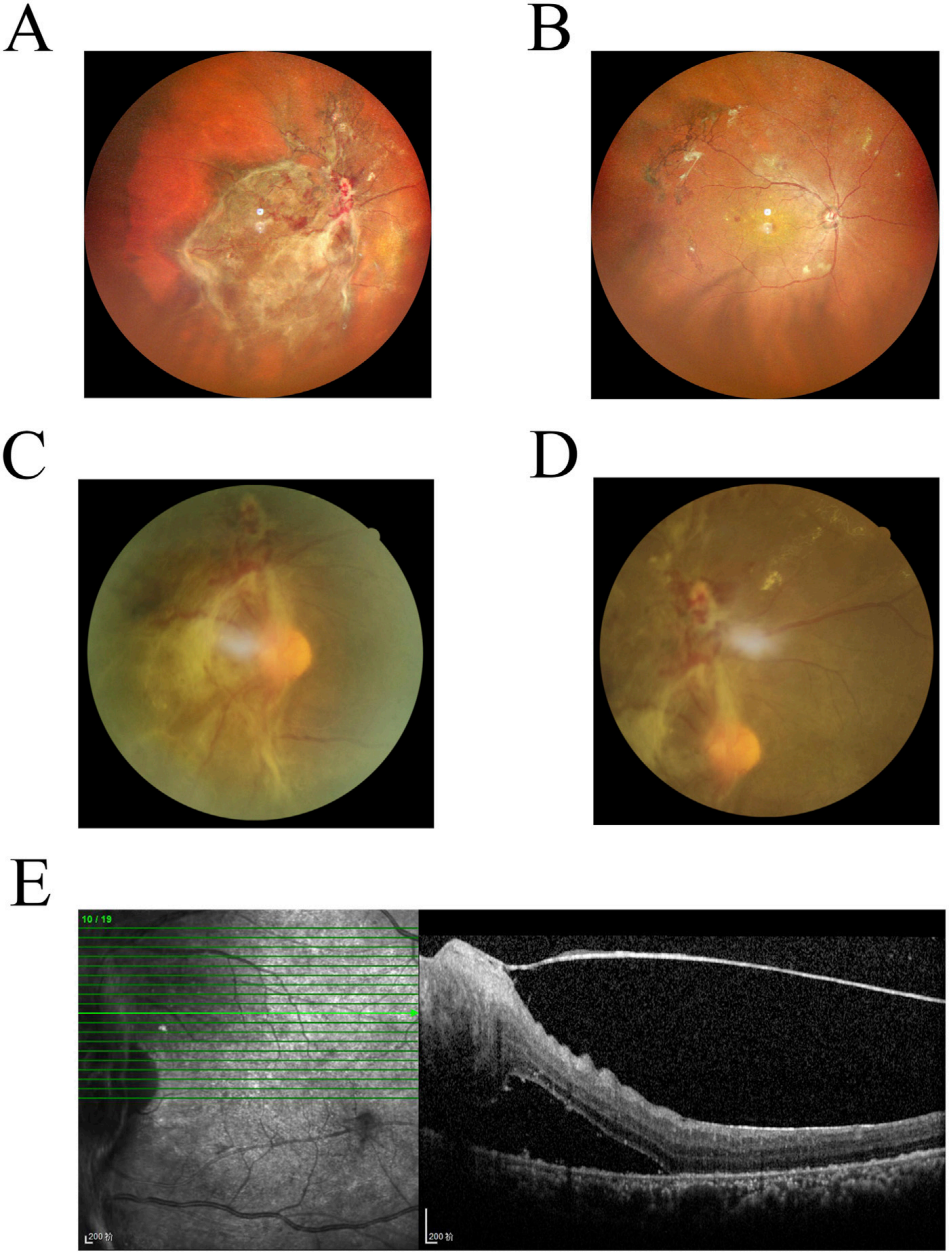
**(A,B)** Laser scanning fundus image of a DR patient. **(C,D)** Fluorescence angiography of the fundus in a DR patient. **(E)** Optical coherence tomography image of a DR patient.

Early-stage DR can be screened through imaging and further confirmed through pathological diagnosis. Figures 7A,B show scanning laser ophthalmoscopy (SLO) images of a DR patient, revealing hemorrhagic spots and proliferative membranes in the fundus. Figures 7C,D present fundus fluorescence angiography images displaying widespread retinal nonperfusion and fluorescent leakage from neovascular formations in both eyes. Figure 7E is an OCT scan that shows macular edema and detachment of the retinal neuroepithelial layer. While imaging technologies such as OCT provide valuable diagnostic insights, their high cost, complex operation, and limited availability hinder large-scale screening. The nanoenzymatic SERS bifunctional detection platform developed in this study offers a more accessible approach for early DR screening.

### 3.7 Analysis of clinical serum samples from DR patients

**FIGURE 8:**
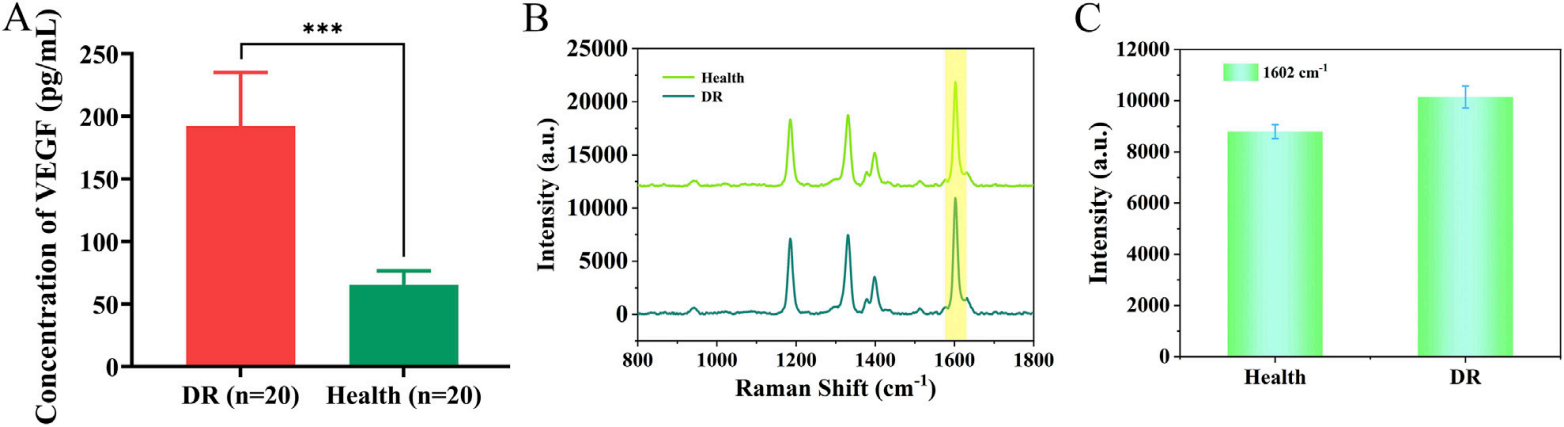
**(A)** Differences in VEGF expression between DR patients and healthy individuals. **(B)** SERS spectra of VEGF detected in serum of healthy individuals and DR patients. **(C)** Histogram of signal intensity at 1,602 cm^−1^ for both groups.

In Figure 8A, we compared the differences between 20 DR patients and 20 healthy individuals by detecting VEGF. The results showed that the VEGF concentration in the DR group was significantly higher than that in the healthy group (P < 0.001). We analyzed serum samples from patients with early-stage DR and healthy individuals using the nanoenzymatic SERS bifunctional detection platform. As shown in Figures 8B,C, a clear difference in SERS signal intensity was observed between the two groups when detecting VEGF. The SERS signal at 1,602 cm^−1^ was substituted into the linear regression equation to calculate the VEGF concentration (ng/mL). To assess accuracy, the results obtained from the SERS platform were compared with those from the ELISA method, which served as the reference standard in this study. As shown in Table 3, the calculated concentrations exhibited relative deviations that remained within an acceptable range, demonstrating the reliability of the detection platform.

**TABLE 3:** Comparison of VEGF expression levels in serum of patients with glycoconjugate network detected by SERS and ELISA.

Sample	SERS (pg/mL)	ELISA (pg/mL)	Relative error (%)
	VEGF	VEGF	VEGF
Healthy	(65.26 ± 17.41)	(60.43 ± 27.35)	7.99
DR	(192.18 ± 66.35)	(204.51 ± 87.86)	−6.02

## 4 Conclusion

In this study, we developed a nanoenzymatic SERS bifunctional detection platform incorporating a competitive recognition strategy based on aptamers, enabling highly specific and ultrasensitive detection of VEGF in serum. The Au@Pd NRs used in the system exhibited strong peroxidase-like catalytic activity along with excellent SERS signal enhancement. By employing an ordered array of Au TOHs with sharp geometric features as the SERS substrate, the tightly packed structure generated numerous electromagnetic “hotspots”, which amplified the SERS signal of catalytically produced oxTMB and significantly improved detection sensitivity and accuracy. The platform demonstrated high reproducibility, operational simplicity, and achieved quantitative detection of VEGF with a limit of detection as low as 0.11 pg/mL. Moreover, results obtained from clinical serum samples were highly consistent with those from the ELISA method, confirming the reliability of the platform. Overall, this detection system holds strong potential for use in identifying low-abundance protein biomarkers and may be applied to early screening of patients with DR.

## Data Availability

The original contributions presented in the study are included in the article/supplementary material, further inquiries can be directed to the corresponding authors.

## References

[B1] Ahuja S. Saxena S. Akduman L. Meyer C. H. Kruzliak P. Khanna V. K. (2019). Serum vascular endothelial growth factor is a biomolecular biomarker of severity of diabetic retinopathy. Int. J. Retina Vitr. 5, 29. 10.1186/s40942-019-0179-6 PMC6771093 31583119

[B2] Bílek R. Zelinka T. Vlček P. Dušková J. Michalský D. Novák K. (2017). Radioimmunoassay of chromogranin A and free metanephrines in diagnosis of pheochromocytoma. Physiol. Res. 66 (Suppl. 3), S397–s408. 10.33549/physiolres.933719 28948824

[B3] Cao Y. Dai Y. Zhang L. Wang D. Yu Q. Hu W. (2022). Serum oncostatin M is a potential biomarker of disease activity and infliximab response in inflammatory bowel disease measured by chemiluminescence immunoassay. Clin. Biochem. 100, 35–41. 10.1016/j.clinbiochem.2021.11.011 34843732

[B4] Chen G. Song J. Zhang H. Jiang Y. Liu W. Zhang W. (2015). Pd nanoparticles encapsulated in magnetic carbon nanocages: an efficient nanoenzyme for the selective detection and multicolor imaging of cancer cells. Nanoscale 7 (34), 14393–14400. 10.1039/c5nr03421c 26248481

[B5] Cheng Q. Yang Y. Peng Y. Liu M. (2020). Pt nanoparticles with high oxidase-like activity and reusability for detection of ascorbic acid. Nanomater. (Basel) 10 (6), 1015. 10.3390/nano10061015 PMC7352317 32466542

[B6] Cheung N. Mitchell P. Wong T. Y. (2010). Diabetic retinopathy. Lancet 376 (9735), 124–136. 10.1016/s0140-6736(09)62124-3 20580421

[B7] de Barros Garcia J. M. B. Isaac D. L. C. Avila M. (2017). Diabetic retinopathy and OCT angiography: clinical findings and future perspectives. Int. J. Retina Vitr. 3, 14. 10.1186/s40942-017-0062-2 PMC5346852 28293432

[B8] Dumestre-Pérard C. Thielens N. M. (2021). Anti-Ficolin-2 and Anti-Ficolin-3 autoantibody detection by ELISA. Methods Mol. Biol. 2227, 121–132. 10.1007/978-1-0716-1016-9_12 33847937

[B9] Egunsola O. Dowsett L. E. Diaz R. Brent M. H. Rac V. Clement F. M. (2021). Diabetic retinopathy screening: a systematic review of qualitative literature. Can. J. Diabetes 45 (8), 725–733.e12. 10.1016/j.jcjd.2021.01.014 33814308

[B10] Hussain I. Rial C. Boza J. Tompkins S. Branen J. Giordano J. (2024). Design of a handheld and portable fluorescence imaging system for quantitative detection of pregnancy-specific biomarkers in cattle. Anal. Bioanal. Chem. 416 (18), 4101–4109. 10.1007/s00216-024-05333-6 38744719

[B11] Khonyoung S. Mangkronkaew P. Klayprasert P. Puangpila C. Palanisami M. Arivazhagan M. (2024). Point-of-Care detection of carcinoembryonic antigen (CEA) using a smartphone-based, label-free electrochemical immunosensor with multilayer CuONPs/CNTs/GO on a disposable screen-printed electrode. Biosens. (Basel) 14 (12), 600. 10.3390/bios14120600 PMC11674700 39727865

[B12] Kropp M. Golubnitschaja O. Mazurakova A. Koklesova L. Sargheini N. Vo T. K. S. (2023). Diabetic retinopathy as the leading cause of blindness and early predictor of cascading complications-risks and mitigation. Epma J. 14 (1), 21–42. 10.1007/s13167-023-00314-8 36866156 PMC9971534

[B13] Lin K. Y. Hsih W. H. Lin Y. B. Wen C. Y. Chang T. J. (2021). Update in the epidemiology, risk factors, screening, and treatment of diabetic retinopathy. J. Diabetes Investig. 12 (8), 1322–1325. 10.1111/jdi.13480 PMC8354492 33316144

[B14] Liu X. Zhou X. Song W. Zeng J. Niu X. Meng R. (2023). The diagnostic value of circulating VEGF in diabetic retinopathy in Asia: a systematic review and meta-analysis. Ophthalmic Epidemiol. 30 (3), 230–238. 10.1080/09286586.2022.2088805 35796414

[B15] Luo M. Zhao F. K. Wang Y. M. Bian J. (2024). Au@Pd nanozyme-mediated catalytic therapy: a novel strategy for targeting tumor microenvironment in cancer treatment. J. Transl. Med. 22 (1), 814. 10.1186/s12967-024-05631-8 39223625 PMC11370004

[B16] Moisoiu V. Iancu S. D. Stefancu A. Moisoiu T. Pardini B. Dragomir M. P. (2021). SERS liquid biopsy: an emerging tool for medical diagnosis. Colloids Surf. B Biointerfaces 208, 112064. 10.1016/j.colsurfb.2021.112064 34517219

[B17] Motz C. T. Chesler K. C. Allen R. S. Bales K. L. Mees L. M. Feola A. J. (2020). Novel detection and restorative levodopa treatment for preclinical diabetic retinopathy. Diabetes 69 (7), 1518–1527. 10.2337/db19-0869 32051147 PMC7306127

[B18] Nilghaz A. Mahdi Mousavi S. Amiri A. Tian J. Cao R. Wang X. (2022). Surface-enhanced raman spectroscopy substrates for food safety and quality analysis. J. Agric. Food Chem. 70 (18), 5463–5476. 10.1021/acs.jafc.2c00089 35471937

[B19] Rodríguez M. L. Pérez S. Mena-Mollá S. Desco M. C. Ortega Á L. (2019). Oxidative stress and microvascular alterations in diabetic retinopathy: future therapies. Oxid. Med. Cell. Longev. 2019, 4940825. 10.1155/2019/4940825 31814880 PMC6878793

[B20] Saeedi P. Petersohn I. Salpea P. Malanda B. Karuranga S. Unwin N. (2019). Global and regional diabetes prevalence estimates for 2019 and projections for 2030 and 2045: results from the international diabetes Federation diabetes atlas, 9(th) edition. Diabetes Res. Clin. Pract. 157, 107843. 10.1016/j.diabres.2019.107843 31518657

[B21] Schreur V. Larsen M. B. Sobrin L. Bhavsar A. R. den Hollander A. I. Klevering B. J. (2022). Imaging diabetic retinal disease: clinical imaging requirements. Acta Ophthalmol. 100 (7), 752–762. 10.1111/aos.15110 35142031

[B22] Song Y. Miao T. Zhang P. Bi C. Xia H. Wang D. (2015). {331}-Faceted trisoctahedral gold nanocrystals: synthesis, superior electrocatalytic performance and highly efficient SERS activity. Nanoscale 7 (18), 8405–8415. 10.1039/c5nr01049g 25877040

[B23] Sun Y. Shi L. Mi L. Guo R. Li T. (2020). Recent progress of SERS optical nanosensors for miRNA analysis. J. Mater Chem. B 8 (24), 5178–5183. 10.1039/d0tb00280a 32432312

[B24] Tang Z. Ali I. Hou Y. Akakuru O. U. Zhang Q. Mushtaq A. (2022). pH-Responsive au@Pd bimetallic core-shell nanorods for enhanced synergistic targeted photothermal-augmented nanocatalytic therapy in the second near-infrared window. J. Mater Chem. B 10 (34), 6532–6545. 10.1039/d2tb01337a 36000458

[B25] Tang Z. Hou Y. Huang S. Hosmane N. S. Cui M. Li X. (2024). Dumbbell-shaped bimetallic AuPd nanoenzymes for NIR-II Cascade catalysis-photothermal synergistic therapy. Acta Biomater. 177, 431–443. 10.1016/j.actbio.2024.01.041 38307478

[B26] Teo Z. L. Tham Y. C. Yu M. Chee M. L. Rim T. H. Cheung N. (2021). Global prevalence of diabetic retinopathy and projection of burden through 2045: systematic review and meta-analysis. Ophthalmology 128 (11), 1580–1591. 10.1016/j.ophtha.2021.04.027 33940045

[B27] Waheed N. K. Rosen R. B. Jia Y. Munk M. R. Huang D. Fawzi A. (2023). Optical coherence tomography angiography in diabetic retinopathy. Prog. Retin Eye Res. 97, 101206. 10.1016/j.preteyeres.2023.101206 37499857 PMC11268430

[B28] Xiang Q. Wang H. Liu S. Zheng Y. Wang S. Zhang H. (2025). Highly sensitive and reproducible SERS substrate based on ordered multi-tipped Au nanostar arrays for the detection of myocardial infarction biomarker cardiac troponin I. Analyst 150 (11), 2239–2250. 10.1039/d5an00171d 40264296

[B29] Xu Z. Xu H. Duan H. Li J. Hu X. Jiang K. (2024). Smartphone-aided fluorescence detection of cardiac biomarker myoglobin by a ratiometric fluorescent AuNCs-QDs nanohybrids probe with high sensitivity. J. Fluoresc. 34 (1), 179–190. 10.1007/s10895-023-03246-8 37166611

[B30] Yang J. Liu Z. (2022). Mechanistic pathogenesis of endothelial dysfunction in diabetic nephropathy and retinopathy. Front. Endocrinol. (Lausanne) 13, 816400. 10.3389/fendo.2022.816400 35692405 PMC9174994

[B31] Zhang Y. Mi X. Tan X. Xiang R. (2019). Recent progress on liquid biopsy analysis using surface-enhanced raman spectroscopy. Theranostics 9 (2), 491–525. 10.7150/thno.29875 30809289 PMC6376192

[B32] Zhang Y. Pang X. Wu D. Ma H. Yan Z. Zhang J. (2016). A robust electrochemiluminescence immunoassay for carcinoembryonic antigen detection based on a microtiter plate as a bridge and au@Pd nanorods as a peroxidase mimic. Analyst 141 (1), 337–345. 10.1039/c5an02053k 26609799

[B33] Zhao C. Jian X. Gao Z. Song Y. Y. (2022). Plasmon-mediated peroxidase-like activity on an asymmetric nanotube architecture for rapid visual detection of bacteria. Anal. Chem. 94 (40), 14038–14046. 10.1021/acs.analchem.2c03471 36170584

[B34] Zhao G. Lochon F. Dembélé K. Florea I. Baron A. Ossikovski R. (2024). Rapid and facile synthesis of gold trisoctahedrons for surface-enhanced raman spectroscopy and refractive index sensing. ACS Appl. Nano Mater 7 (5), 5598–5609. 10.1021/acsanm.4c00455 38481750 PMC10928655

